# Flavonoid-rich dietary patterns and the risk of incident hearing loss: evidence from the UK Biobank cohort

**DOI:** 10.1016/j.jnha.2026.100891

**Published:** 2026-06-03

**Authors:** Youngji Han, Kyu-Yup Lee, Jeong Heon Lee, Incheol Seo, Da Jung Jung

**Affiliations:** a Bio-Medical Research Institute, Kyungpook National University Hospital, Daegu, Republic of Korea; b Department of Otorhinolaryngology–Head and Neck Surgery, School of Medicine, Kyungpook National University, Daegu, Republic of Korea; c Department of Artificial Intelligence, Kyungpook National University, Republic of Korea; d Department of Immunology, School of Medicine, Kyungpook National University, Daegu, Republic of Korea

**Keywords:** Flavonoids, Diet quality, Hearing loss, Inflammation, UK Biobank, Cohort study

## Abstract

**Background:**

Hearing loss is a major sensory impairment linked to oxidative stress and inflammation. Flavonoids possess antioxidant and anti-inflammatory properties, but their longitudinal association with incident hearing loss is unclear. We examined the association between a Flavonoid Diet Score (FDS) and incident hearing loss in the UK Biobank, and explored potential mediation by systemic inflammation.

**Methods:**

We included 55,859 participants without baseline hearing loss who completed 24-h dietary recalls. FDS was calculated from flavonoid-rich food intake and categorized into quartiles. Incident hearing loss was identified using ICD-10 codes (H90, H91) from linked hospital and death records. Cox proportional hazards models estimated hazard ratios (HRs) and 95% confidence intervals (CIs). Mediation analyses assessed whether inflammatory biomarkers (C-reactive protein [CRP], glycoprotein acetyls, neutrophil count/percentage, and leukocyte count) explained the association.

**Results:**

During 613,590 person-years of follow-up, 1,681 incident cases of hearing loss occurred. After adjusting for demographic, lifestyle, and cardiometabolic factors, higher FDS was associated with a reduced risk of hearing loss (HR for Q4 vs. Q1 = 0.84, 95% CI: 0.73–0.96; P for trend = 0.021). Among the evaluated biomarkers, CRP significantly mediated this association (average causal mediation effect = −0.0079, P = 0.010), accounting for 4.8% of the total effect. Other inflammatory markers were not significant mediators.

**Conclusions:**

Higher adherence to a flavonoid-rich diet was independently associated with a lower risk of incident hearing loss, an effect partially mediated by reduced systemic inflammation (CRP). Promoting dietary flavonoids may serve as a beneficial nutritional strategy to preserve auditory aging.

## 1 Introduction

Hearing loss is one of the most prevalent sensory impairments globally, affecting more than 400 million people and substantially contributing to disability, cognitive decline, and reduced quality of life in aging populations [1,2]. Despite its growing public health burden, preventive strategies remain limited, and most existing interventions focus on hearing rehabilitation rather than primary prevention. Recent research has suggested that cochlear aging involves not only mechanical damage from noise exposure but also systemic factors such as oxidative stress, vascular dysfunction, and chronic inflammation that compromise cochlear microcirculation and hair cell survival [3,4]. These mechanisms resemble those observed in cardiovascular and metabolic aging, suggesting potentially shared preventive pathways [5].

Flavonoids are naturally occurring bioactive compounds found in plant-based foods, including berries, citrus fruits, tea, and dark-colored vegetables [6]. They exhibit antioxidant, anti-inflammatory, and vasoprotective properties that enhance endothelial function, regulate nitric oxide bioavailability, and mitigate lipid peroxidation and inflammatory signaling [7,8]. Through these mechanisms, flavonoids may help preserve microvascular and neuronal integrity. Previous epidemiological studies have demonstrated that higher dietary flavonoid intake is associated with a lower risk of cardiovascular disease, type 2 diabetes, and cognitive decline [[9], [10], [11], [12], [13], [14]]; however, few studies have investigated whether flavonoids may also protect against sensory deterioration, such as age-related hearing loss.

Emerging evidence from population-based cohorts suggests that specific flavonoid subclasses, including flavonols, flavan-3-ols, and anthocyanins, may be inversely associated with the long-term incidence of hearing loss and tinnitus. However, findings remain inconsistent, and the potential biological mediators underlying these associations have not been fully explored [15,16]. Therefore, this study aimed to investigate the relationship between a flavonoid-rich dietary pattern, as quantified by the Flavonoid Diet Score (FDS), and the risk of incident hearing loss in a large population-based cohort. We further explored whether inflammatory biomarkers mediate this association, hypothesizing that higher flavonoid intake is associated with a lower risk of hearing loss, partly through reductions in systemic inflammation.

## 2 Methods

### 2.1 Study population

Figure 1 presents the flow chart of participant selection. This prospective cohort study was conducted using data from the UK Biobank, a large-scale population-based cohort that recruited 501,962 participants aged 40–69 years across the United Kingdom between 2006 and 2010. Detailed information on the design, rationale, and data collection procedures of the UK Biobank has been described previously [17]. Among the total 501,962 participants, those with incomplete baseline dietary questionnaire data were first excluded (N = 431,327). We then excluded participants with prevalent hearing loss at baseline (N = 1,004), those who answered “No” to the 24 -h recall question, indicating that the reported diet was not representative of their usual intake (Data-Field 100020; N = 12,641), and those with implausible total energy intake values (<3,347 or >17,573 kJ/day for men; <2,092 or >14,644 kJ/day for women) (N = 1,131) [18]. After applying these exclusion criteria, a total of 55,859 participants with complete and plausible dietary data were included in the final analytical cohort. All participants provided informed consent, and the UK Biobank study received ethical approval from the North West Multi-Centre Research Ethics Committee (REC reference 11/NW/0382).

### 2.2 Exposure assessment

Dietary flavonoid intake was assessed using the Oxford WebQ 24-h dietary recall questionnaire, which was administered on up to five separate occasions between 2009 and 2012. This web-based tool recorded all foods and beverages consumed during the previous day and was validated against interviewer-administered 24-h recalls and biomarkers of intake [19].

The Flavonoid Diet Score (FDS) was constructed to represent adherence to a flavonoid-rich dietary pattern. The validity and reproducibility of this scoring system have been previously demonstrated in large cohort settings [9,11,20]. The score was derived from the relative intake of major flavonoid-contributing food groups, including tea, citrus fruits, apples, berries, and dark-colored vegetables (Supplementary Table S1). Each participant’s consumption of these food groups was quantified from the nutrient composition database and standardized for total energy intake. Participants were categorized into quartiles (Q1–Q4) based on the distribution of their total flavonoid intake, with Q1 indicating the lowest and Q4 the highest adherence to a flavonoid-rich diet.

### 2.3 Outcome assessment

The primary outcome was incident hearing loss, defined as the first recorded diagnosis of ICD-10 codes H90 (Conductive and sensorineural hearing loss) or H91 (Other and unspecified hearing loss) during follow-up. Data were obtained from linked hospital inpatient records, death registries, and self-reported health outcomes in the UK Biobank.

Participants with a diagnosis of hearing loss before baseline were excluded. Follow-up time was calculated from baseline assessment to the first occurrence of hearing loss, death, loss to follow-up, or end of data availability (October 2024), whichever came first.

A binary outcome variable was coded as 1 for participants who developed hearing loss and 0 for those who did not experience an event during follow-up. Total person-years were accumulated accordingly.

### 2.4 Covariates

Covariates were selected based on prior literature on dietary factors and auditory health. Adjustments included demographic variables (age, sex, race/ethnicity), socioeconomic status (Townsend Deprivation Index), lifestyle factors (smoking status, alcohol consumption, physical activity measured in metabolic equivalent task [MET] hours per week), and body mass index (BMI). Preexisting comorbidities, including dyslipidemia, diabetes mellitus, cardiovascular disease (CVD), and hypertension, were defined by hospital records and verified diagnoses. Tinnitus and depression were identified from self-report data. Noise exposure variables included occupational noise and exposure to frequent loud music, both self-reported at baseline.

Missing categorical covariates were imputed using mode imputation, and missing continuous variables were replaced with the sample median. The exact number and proportion of missing values for each variable prior to imputation are detailed in Supplementary Table S2.

### 2.5 Measurement of inflammatory biomarkers for mediation analysis

Inflammatory biomarkers were measured at baseline as part of the UK Biobank Biomarker Enhancement Project. Serum C-reactive protein (CRP) was quantified using a high-sensitivity immunoturbidimetric assay (Beckman Coulter AU5800, UK) with an analytical range of 0.08–80 mg/L. Additional biomarkers, including glycoprotein acetyls (GlycA) and neutrophil count, were measured from EDTA plasma using high-throughput NMR spectroscopy (Nightingale Health platform).

Haematological biomarkers, including neutrophil count, neutrophil percentage, and leukocyte (white blood cell) count, were obtained from whole-blood assays conducted at baseline as part of the UK Biobank Haematology Data Release (Category 100081). Whole blood samples were collected in 4 mL EDTA vacutainers and analyzed within 24 h of collection at the UK Biobank central laboratory. Analyses were performed using Beckman Coulter LH750 haematology analyzers, which provide automated quantitative measurement of complete blood count (CBC) and differential leukocyte parameters. The LH750 analyzer utilizes the Coulter impedance principle, combined with Volume, Conductivity, and Scatter (VCS) technology, to distinguish cell populations and quantify differential counts.

### 2.6 Statistical analysis

Baseline characteristics across quartiles of the Flavonoid Diet Score (FDS) were summarized as means (standard deviation) for continuous variables and counts (percentage) for categorical variables. Linear regression and logistic regression were used to test for linear trends across quartiles of FDS.

Cox proportional hazards regression models were applied to estimate hazard ratios (HRs) and 95% confidence intervals (CIs) for incident hearing loss across quartiles of FDS, using Q1 as the reference group. Three sequential models were fitted: (1) univariable; (2) adjusted for age and sex; and (3) multivariable, additionally adjusting for lifestyle, socioeconomic, and clinical covariates. A test for linear trend was conducted by modeling the median value of each quartile as a continuous variable.

Subgroup analyses were conducted according to age (<60 or ≥60 years), sex, BMI, Townsend Deprivation Index, smoking, alcohol use, dyslipidemia, CVD, and noise exposure. Interaction terms were tested using likelihood ratio tests.

Sensitivity analyses were performed by sequentially excluding participants with significant comorbidities (hypertension, diabetes, CVD, or dyslipidemia) or known noise exposure to assess the robustness of the associations.

To assess mediation, we employed a counterfactual-based causal mediation analysis framework, with CRP and GlycA as mediators. The indirect (average causal mediation effect, ACME), direct (average direct effect, ADE), and total effects were estimated using linear regression for the mediator and Cox models for the outcome. The proportion mediated was calculated as ACME divided by the total effect. Confidence intervals were derived from nonparametric bootstrapping with 1,000 resamples.

All statistical analyses were performed using Python version 3.9.5 (Python Software Foundation, Wilmington, DE, USA). Two-sided P values <0.05 were considered statistically significant.

## 3 Results

### 3.1 Baseline characteristics according to quartiles of the flavonoid diet score

In this cohort (n = 55,859), participants with higher Flavonoid Diet Scores (FDS) tended to be older, more likely to be women, and had a slightly lower mean BMI, lower levels of area-based deprivation (more negative Townsend Deprivation Index), and higher physical activity levels (MET) (all *P* < 0.001) (Table 1). The prevalence of current smoking decreased markedly across FDS quartiles (from 11.6% in Q1 to 5.6% in Q4; *P* < 0.001), whereas alcohol consumption remained similar across groups (approximately 92–93%; *P* = 0.080). Regarding clinical conditions, the prevalence of cardiovascular disease (CVD) and dyslipidemia was modestly lower in higher FDS quartiles (*P* < 0.001 and *P* = 0.026, respectively), while hypertension showed no significant differences (*P* = 0.481). Diabetes prevalence also showed a decreasing but nonsignificant trend (*P* = 0.089). Higher FDS was associated with fewer self-reported hearing difficulties and difficulty hearing in background noise (*P* = 0.044 and *P* < 0.001, respectively), whereas hearing aid use and cochlear implant rates were comparable across groups. Participants with higher FDS also reported less occupational and recreational noise exposure (*P* = 0.001 and *P* < 0.001, respectively). Ethnic distribution differed slightly across quartiles, with a greater proportion of White participants in the higher FDS categories.

**Table 1:** Baseline characteristics of participants according to quartiles of the Flavonoid diet Score.

Variable	Q1 (n = 13,965)	Q2 (n = 13,965)	Q3 (n = 13,964)	Q4 (n = 13,965)	Overall (n = 55,859)	P
**FDS,** mean (SD)	0.00 (0.00)	0.55(0.50)	1.40(0.49)	3.14(1.22)	1.27(1.38)	<0.001
Demographics and lifestyle characteristics						
Age, mean (SD)	55.69 (8.35)	56.33 (8.16)	56.89 (8.04)	56.85 (7.93)	56.44 (8.14)	<0.001
Sex, No.(%)						
Female	6,984 (50.0)	7,370 (52.8)	7,778 (55.7)	8,327 (59.6)	30,459 (54.5)	<0.001
Male	6,981 (50.0)	6,595 (47.2)	6,186 (44.3)	5,638 (40.4)	25,400 (45.5)	<0.001
Race and ethnicity, No. (%)						
Asian	463 (3.3)	387 (2.8)	331 (2.4)	298 (2.1)	1,479 (2.6)	<0.001
Black	334 (2.4)	278 (2.0)	239 (1.7)	241 (1.7)	1,092 (2.0)	<0.001
Mixed	111 (0.8)	105 (0.8)	80 (0.6)	99 (0.7)	395 (0.7)	<0.001
Other	119 (0.9)	115 (0.8)	113 (0.8)	153 (1.1)	500 (0.9)	<0.001
Unknown	50 (0.4)	56 (0.4)	52 (0.4)	66 (0.5)	224 (0.4)	<0.001
White	12,884 (92.3)	13,021 (93.2)	13,146 (94.1)	13,106 (93.8)	52,157 (93.4)	<0.001
BMI, mean (SD)	27.44 (4.83)	27.12 (4.71)	26.96 (4.62)	26.93 (4.76)	27.11 (4.74)	<0.001
Smoking status, No. (%)	1,609 (11.5)	1,257 (9.0)	928 (6.6)	774 (5.5)	4,568 (8.2)	<0.001
Alcohol status, No. (%)	12,873 (92.2)	12,881 (92.2)	12,981 (93.0)	12,893 (92.3)	51,628 (92.4)	0.080
Townsend deprivation index, mean (SD)	−1.09 (2.93)	−1.27 (2.84)	−1.43 (2.76)	−1.42 (2.77)	−1.30 (2.83)	<0.001
MET, mean (SD)	42.39 (43.49)	44.33 (43.68)	45.24 (42.27)	48.72 (44.58)	45.21 (43.58)	<0.001
Health condition						
Dyslipidemia, No. (%)	3,633 (26.0)	3,462 (24.8)	3,489 (25.0)	3,429 (24.6)	14,013 (25.1)	0.026
Diabetes, No. (%)	1,319 (9.4)	1,287 (9.2)	1,230 (8.8)	1,211 (8.7)	5,047 (9.0)	0.089
CVD, No. (%)	1,072 (7.7)	950 (6.8)	925 (6.6)	868 (6.2)	3,815 (6.8)	<0.001
Hypertension, No. (%)	5,323 (38.1)	5,307 (38.0)	5,232 (37.5)	5,220 (37.4)	21,082 (37.7)	0.481
Hearing and Noise Exposure						
Hearing difficulty/ problems, No. (%)	3,434 (24.6)	3,405 (24.4)	3,341 (23.9)	3,256 (23.3)	13,436 (24.1)	0.044
Hearing difficulty/ problems with background noise, No. (%)	4,992 (35.7)	4,894 (35.0)	4,755 (34.1)	4,519 (32.4)	19,160 (34.3)	<0.001
Hearing aid user, No. (%)	307 (2.2)	334 (2.4)	297 (2.1)	278 (2.0)	1,216 (2.2)	0.136
Cochlear implant, No. (%)	5 (0.0)	11 (0.1)	9 (0.1)	8 (0.1)	33 (0.1)	0.517
Noisy workplace, No. (%)	3,956 (28.3)	3,873 (27.7)	3,829 (27.4)	3,698 (26.5)	15,356 (27.5)	0.001
Loud music exposure, No. (%)	1,890 (13.5)	1,733 (12.4)	1,487 (10.6)	1,598 (11.4)	6,708 (12.0)	<0.001

Values are presented as mean (standard deviation) for continuous variables and number (percentage) for categorical variables. P values were calculated using analysis of variance (ANOVA) for continuous variables and chi-square (χ^2^) tests for categorical variables to assess differences across quartiles of the Flavodiet Score (FDS); The Townsend deprivation index (TDI) reflects area-level socioeconomic deprivation, with higher values indicating greater deprivation; MET represents total metabolic-equivalent task hours per week; Hearing difficulty and noise-exposure variables were self-reported at baseline; FDS quartiles (Q1–Q4) correspond to increasing levels of flavonoid-rich food consumption derived from multiple Oxford WebQ 24-h dietary assessments; All tests were two-sided, and a P value < 0.05 was considered statistically significant.

BMI, body mass index; CVD, cardiovascular disease; FDS, Flavodiet Score; MET, metabolic-equivalent task; SD, standard deviation; TDI, Townsend deprivation index.

### 3.2 Association between flavonoid diet score and incident hearing loss

During a total follow-up of 613,590 person-years, 1,681 participants developed incident hearing loss (Table 2). The incidence rate of hearing loss decreased progressively across FDS quartiles, from 2.80 per 1,000 person-years in Q1 to 2.47 per 1,000 person-years in Q4. In Cox proportional hazards models (Table 3), higher FDS quartiles were associated with a lower risk of incident hearing loss. In the univariable model, participants in Q4 had a hazard ratio (HR) of 0.87 (95% CI, 0.76–1.00) compared with those in Q1. After adjustment for age and sex, the inverse association became slightly stronger (HR = 0.83; 95% CI, 0.72–0.95). This relationship remained statistically significant in the fully multivariable-adjusted model (HR = 0.84; 95% CI, 0.73–0.96). Collectively, these results indicate that greater adherence to a flavonoid-rich dietary pattern was independently associated with a reduced risk of hearing loss.

**Table 2:** Incidence rates of hearing loss according to quartiles of the Flavonoid Diet Score.

Flavonoid Diet Score (Quartiles)	Person-Years	Events	Incidence Rate (per 1,000 PY)
Q1	150,994.6	423	2.801426
Q2	152,085.1	440	2.893116
Q3	154,471.8	433	2.803101
Q4	156,039.0	385	2.467332

**Table 3:** Hazard ratios (95% confidence intervals) for incident hearing loss according to quartiles of the Flavonoid Diet Score.

	Flavonoid Diet Score (Quartiles)			
Model	Q1	Q2	Q3	Q4
Univariable	1 [Reference]	1.03(0.90−1.18)	0.99(0.87−1.14)	0.87(0.76−1.00)
Sex and age adjusted	1 [Reference]	0.99(0.87−1.13)	0.93(0.82−1.07)	0.83(0.72−0.95)
Multivariable adjusted^a^	1 [Reference]	1.00(0.88−1.14)	0.95(0.83−1.09)	0.84(0.73−0.96)

a Adjusted for age, sex, BMI, smoking status, alcohol intake, Townsend deprivation index, physical activity (MET), dyslipidemia, diabetes, cardiovascular disease, and hypertension.

### 3.3 Subgroup and sensitivity analyses of the association between flavonoid diet score and hearing loss

Subgroup analyses showed that the inverse association between the Flavonoid Diet Score (FDS) and incident hearing loss was generally consistent across demographic and clinical strata (Table 4). The protective association of higher FDS (Q4 vs Q1) was slightly stronger among participants aged <60 years (HR = 0.85, 95% CI 0.72–1.00), males (HR = 0.78, 95% CI 0.64–0.95), those with lower deprivation index (HR = 0.78, 95% CI 0.64–0.95), and participants with BMI below the median (HR = 0.80, 95% CI 0.66–0.96). A similar pattern was observed among noncurrent smokers (HR = 0.80, 95% CI 0.70–0.93) and current alcohol users (HR = 0.85, 95% CI 0.74–0.99). The inverse association was also evident in participants with baseline dyslipidemia (HR = 0.75, 95% CI 0.60–0.93) and cardiovascular disease (HR = 0.68, 95% CI 0.47–0.98). No significant interactions were detected between FDS and age, sex, BMI, or lifestyle factors (all P for interaction > 0.05).

**Table 4:** Subgroup analyses of the association between the Flavonoid Diet Score (FDS) and incident hearing loss.

			Flavonoid Diet Score (Quartiles), HR (95%CI)a			
Subgroups		N	Q1	Q2	Q3	Q4
			(N = 273,014)	(N = 137,974)	(N = 57,242)	(N = 23,938)
Age, y	< 60	31405	1 [Reference]	0.99(0.84−1.16)	0.99(0.84−1.16)	0.85(0.72−1.00)
	>= 60	24454	1 [Reference]	1.03(0.82−1.31)	0.89(0.69−1.14)	0.82(0.64−1.06)
Sex	Female	30459	1 [Reference]	1.12(0.92−1.37)	0.96(0.78−1.18)	0.91(0.74−1.11)
	Male	25400	1 [Reference]	0.91(0.76−1.09)	0.95(0.80−1.14)	0.78(0.64−0.95)
Race and ethnicity	White	52157	1 [Reference]	1.00(0.87−1.14)	0.94(0.82−1.08)	0.83(0.72−0.95)
	Othersa	3702	1 [Reference]	0.97(0.48−1.96)	1.20(0.61−2.36)	1.04(0.52−2.08)
Townsend Deprivation Index score, quintile	<Median	27930	1 [Reference]	1.05(0.87−1.26)	0.84(0.69−1.02)	0.78(0.64−0.95)
	≥Median	27839	1 [Reference]	0.95(0.78−1.16)	1.08(0.89−1.30)	0.91(0.74−1.10)
BMI	<Median	27851	1 [Reference]	0.92(0.77−1.09)	0.91(0.76−1.09)	0.80(0.66−0.96)
	≥Median	27864	1 [Reference]	1.15(0.93−1.42)	1.04(0.85−1.29)	0.91(0.73−1.13)
Smoking status	Noncurrent	51127	1 [Reference]	0.94(0.81−1.08)	0.92(0.80−1.05)	0.80(0.70−0.93)
	Current	4568	1 [Reference]	1.91(1.18−3.10)	1.39(0.81−2.40)	1.24(0.69−2.25)
Alcohol status	Noncurrent	4169	1 [Reference]	1.00(0.64−1.55)	0.98(0.63−1.53)	0.71(0.44−1.14)
	Current	51628	1 [Reference]	1.00(0.87−1.15)	0.95(0.82−1.09)	0.85(0.74−0.99)
Baseline dyslipidemia	Yes	14013	1 [Reference]	0.87(0.70−1.07)	1.05(0.86−1.29)	0.75(0.60−0.93)
	No	41846	1 [Reference]	1.10(0.92−1.31)	0.88(0.74−1.06)	0.91(0.76−1.08)
Baseline CVD	Yes	3815	1 [Reference]	0.92(0.66−1.29)	0.76(0.54−1.09)	0.68(0.47−0.98)
	No	52044	1 [Reference]	1.02(0.88−1.18)	0.99(0.86−1.15)	0.87(0.75−1.01)

^a Adjusted for age, sex, BMI, smoking status, alcohol intake, Townsend deprivation index, physical activity (MET), dyslipidemia, diabetes, cardiovascular disease, and hypertension.

### 3.4 Mediation analysis of the association between flavonoid diet score and hearing loss

To explore potential biological mechanisms linking flavonoid intake with hearing loss, mediation analyses were conducted using circulating inflammatory biomarkers as potential mediators (Table 5). Among the evaluated biomarkers, C-reactive protein (CRP) demonstrated a statistically significant mediating effect on the association between a higher Flavonoid Diet Score (FDS) and a reduced risk of hearing loss. Specifically, in the comparison of Q4 versus Q1, the average causal mediation effect (ACME) of CRP was −0.0079 (95% CI, −0.0154 to −0.0012; *P* = 0.010), and the average direct effect (ADE) was −0.1568 (95% CI, −0.3063 to −0.0121; *P* = 0.018), with a total effect of −0.1647 (*P* = 0.016). Approximately 4.8% of the total effect of higher flavonoid intake on hearing loss risk was mediated through CRP (*P* = 0.025), indicating a modest but significant contribution of systemic inflammation to this relationship. By contrast, glycoprotein acetyls, neutrophil count, neutrophil percentage, and leukocyte count did not show significant mediation effects (*P* > 0.05 for all comparisons). These findings suggest that the protective association between higher flavonoid consumption and lower risk of hearing loss may be partially explained by reductions in systemic inflammation, as reflected by lower circulating CRP levels.

**Table 5:** Mediation analyses of inflammatory biomarkers in the association between the Flavonoid Diet Score and incident hearing loss.

Mediator	Outcome Group	Effect	Estimate	95% CIL	95% CIH	p-value
Glycoprotein Acetyls (n = 29,491)	Q2 (n = 7,367)	Average Causal Mediation Effect	−0.0006	−0.0028	0.001	0.2617
		Average Direct Effect	−0.0026	−0.1944	0.1897	0.4775
		Total Effect	−0.0032	−0.1953	0.1882	0.4765
		Proportion Mediated	0.1832	−0.1374	0.1027	0.4815
	Q3 (n = 7,435)	Average Causal Mediation Effect	−0.0016	−0.0056	0.0017	0.1718
		Average Direct Effect	0.0723	−0.105	0.2598	0.2148
		Total Effect	0.0707	−0.1088	0.2586	0.2188
		Proportion Mediated	−0.0222	−0.2278	0.1767	0.2977
	Q4 (n = 7,218)	Average Causal Mediation Effect	−0.0031	−0.0105	0.0041	0.1958
		Average Direct Effect	−0.1209	−0.3063	0.0748	0.1179
		Total Effect	−0.124	−0.3124	0.0705	0.1109
		Proportion Mediated	0.025	−0.1822	0.3287	0.2617
C-reactive protein (n = 52,478)	Q2 (n = 13,153)	Average Causal Mediation Effect	−0.0012	−0.0035	0.0001	0.047
		Average Direct Effect	0.0124	−0.131	0.1517	0.4346
		Total Effect	0.0112	−0.131	0.1506	0.4416
		Proportion Mediated	−0.1053	−0.2587	0.2447	0.4456
	Q3 (n = 13,127)	Average Causal Mediation Effect	−0.0045	−0.0088	−0.0008	0.012
		Average Direct Effect	−0.0305	−0.1667	0.0955	0.3077
		Total Effect	−0.035	−0.1713	0.0911	0.2917
		Proportion Mediated	0.1286	−0.6882	0.6734	0.2987
	Q4 (n = 13,080)	Average Causal Mediation Effect	−0.0079	−0.0154	−0.0012	0.01
		Average Direct Effect	−0.1568	−0.3063	−0.0121	0.018
		Total Effect	−0.1647	−0.3112	−0.0196	0.016
		Proportion Mediated	0.048	0.0011	0.2144	0.025
Neutrophill count (n = 53,366)	Q2 (n = 13,371)	Average Causal Mediation Effect	−0.0002	−0.0014	0.0007	0.3506
		Average Direct Effect	0.0104	−0.1296	0.145	0.4416
		Total Effect	0.0102	−0.1297	0.1451	0.4406
		Proportion Mediated	−0.0184	−0.0875	0.0574	0.4785
	Q3 (n = 13,363)	Average Causal Mediation Effect	−0.0011	−0.0035	0.0011	0.1518
		Average Direct Effect	−0.0416	−0.1859	0.0865	0.2997
		Total Effect	−0.0427	−0.186	0.0848	0.2967
		Proportion Mediated	0.025	−0.1919	0.25	0.3656
	Q4 (n = 13,312)	Average Causal Mediation Effect	−0.0018	−0.0049	0.0016	0.1449
		Average Direct Effect	−0.1446	−0.2852	−0.0117	0.016
		Total Effect	−0.1464	−0.2866	−0.0138	0.016
		Proportion Mediated	0.0125	−0.0155	0.0847	0.1538
Neutrophill percentage (n = 53,366)	Q2 (n = 13,371)	Average Causal Mediation Effect	−0.0004	−0.0017	0.0005	0.2418
		Average Direct Effect	0.0104	−0.1304	0.1449	0.4426
		Total Effect	0.01	−0.1309	0.1449	0.4436
		Proportion Mediated	−0.0382	−0.1316	0.0715	0.4785
	Q3 (n = 13,363)	Average Causal Mediation Effect	−0.0007	−0.0026	0.0004	0.1359
		Average Direct Effect	−0.0422	−0.186	0.0857	0.2977
		Total Effect	−0.0429	−0.1858	0.0847	0.2937
		Proportion Mediated	0.0173	−0.1327	0.1522	0.3367
	Q4 (n = 13,312)	Average Causal Mediation Effect	−0.0013	−0.0037	0.0005	0.0959
		Average Direct Effect	−0.1455	−0.2861	−0.0126	0.016
		Total Effect	−0.1467	−0.2866	−0.0151	0.016
		Proportion Mediated	0.0086	−0.0072	0.0539	0.1089
Leukocyte count (n = 53,611)	Q2 (n = 13,424)	Average Causal Mediation Effect	−0.0002	−0.0009	0.0005	0.3247
		Average Direct Effect	0.0116	−0.1358	0.1437	0.4276
		Total Effect	0.0114	−0.1357	0.1432	0.4296
		Proportion Mediated	−0.0142	−0.0608	0.0472	0.4715
	Q3 (n = 13,422)	Average Causal Mediation Effect	−0.0005	−0.0018	0.0012	0.2468
		Average Direct Effect	−0.0427	−0.1836	0.0915	0.2787
		Total Effect	−0.0431	−0.1832	0.091	0.2757
		Proportion Mediated	0.0108	−0.1139	0.1028	0.3816
	Q4 (n = 13,389)	Average Causal Mediation Effect	−0.0009	−0.0029	0.0023	0.2607
		Average Direct Effect	−0.1486	−0.3004	−0.0065	0.022
		Total Effect	−0.1495	−0.3007	−0.0072	0.02
		Proportion Mediated	0.0057	−0.0263	0.052	0.2657

Models were adjusted for age, sex, BMI, smoking status, alcohol intake, Townsend deprivation index, physical activity (MET), dyslipidemia, diabetes, cardiovascular disease, and hypertension.

CIL, lower bound of the 95% confidence interval; CIH, upper bound of the 95% confidence interval.

## 4 Discussion

In this large population-based cohort, greater adherence to a flavonoid-rich dietary pattern, represented by a higher FDS, was independently associated with a lower risk of incident hearing loss. The inverse association remained robust after comprehensive adjustment for demographic, lifestyle, and cardiometabolic risk factors, suggesting that flavonoids may exert a direct protective effect on auditory function through biological mechanisms that extend beyond confounding lifestyle behaviors. Furthermore, mediation analysis indicated that systemic inflammation, reflected by circulating CRP levels, partially explained this association, supporting an anti-inflammatory mechanism underlying the observed relationship. Furthermore, the incidence rate of hearing loss observed in our study (2.47–2.89 per 1,000 person-years) is highly comparable to those reported in recent epidemiological studies utilizing the UK Biobank population. For instance, a recent study by Han et al. reported incidence rates ranging from 3.37 to 4.33 per 1,000 person-years using identical diagnostic criteria [21] The slightly lower incidence in our study is anticipated, as our analysis was restricted to the sub-cohort that completed the Oxford WebQ 24 -h dietary recall (N = 55,859), compared to the broader cohort assessed via baseline touchscreen questionnaires (N > 490,000). Because participants who complete detailed dietary recalls often reflect a more health-conscious demographic, a marginally lower incidence rate is expected. Taking this methodological difference into account, our incidence rates demonstrate robust consistency with the existing literature, highlighting the reliability of our outcome ascertainment.

Our findings reinforce and expand previous epidemiologic observations showing that diets rich in flavonoids and plant-derived antioxidants are inversely associated with age-related hearing decline [15,22]. Prior cohort studies, including the Blue Mountains Hearing Study, have demonstrated that higher intake of flavonoid subclasses such as flavonols, flavan-3-ols, and anthocyanins is linked to a reduced 10-year incidence of age-related hearing loss and tinnitus [15,16]. Mechanistically, flavonoids exert a broad spectrum of biological effects that converge on oxidative stress and vascular inflammation, two hallmarks of cochlear aging [23]. They enhance endothelial nitric oxide bioavailability, attenuate oxidative injury within the stria vascularis, and preserve cochlear microcirculation [24], thereby maintaining the delicate ionic and metabolic balance essential for hair cell survival. These mechanistic insights are consistent with our findings that higher adherence to a flavonoid-rich dietary pattern was associated with a lower risk of hearing loss, suggesting that the antioxidant and vascular-protective properties of flavonoids may underlie their auditory benefits. Experimental research provides complementary evidence at the molecular level. In animal and in vitro models, flavonoids such as quercetin, epigallocatechin gallate, and hesperetin have been shown to protect auditory hair cells against cisplatin-induced ototoxicity, aminoglycoside exposure, and noise-induced trauma [[25], [26], [27]]. These compounds modulate key antioxidant pathways, including Nrf2 and SIRT1, stabilize the mitochondrial membrane potential, and suppress lipid peroxidation within cochlear tissues [28]. The present mediation analysis supports the hypothesis that systemic inflammation, indexed by CRP, acts as a mechanistic bridge linking dietary flavonoid intake with auditory resilience. Given that elevated CRP predicts accelerated hearing decline and cochlear vascular compromise, our findings lend biological plausibility to an anti-inflammatory model of auditory protection.

From a clinical and preventive perspective, these results carry significant public health implications. Hearing loss remains one of the major causes of disability among older adults, affecting more than 400 million people globally and contributing to cognitive decline, depression, and social isolation [29]. Despite its widespread burden, effective pharmacological prevention strategies are still lacking [30]. From a translational standpoint, these findings bridge the gap between nutritional epidemiology and auditory medicine by identifying systemic inflammation as a modifiable biological pathway. This suggests that dietary counseling or targeted nutritional interventions may serve as feasible adjunctive strategies in clinical hearing preservation programs. Our findings suggest that dietary modification, particularly through greater consumption of flavonoid-rich foods such as berries, citrus fruits, green tea, and dark-colored vegetables, may offer a practical and low-cost approach to mitigating sensory aging. The consistent inverse associations observed across demographic and metabolic subgroups indicate that this relationship is broadly generalizable across populations. Notably, stronger associations were observed among men, younger individuals, and those with metabolic dysregulation, suggesting that individuals under greater oxidative or inflammatory stress may experience greater benefits from flavonoid intake [15].

Beyond auditory health, these observations fit within a broader physiological framework wherein flavonoids confer systemic vascular and metabolic protection. Numerous studies have demonstrated that flavonoid intake improves endothelial reactivity, enhances insulin sensitivity, lowers blood pressure, and mitigates chronic low-grade inflammation [[31], [32], [33]]. These shared biological mechanisms, centered on the maintenance of microvascular integrity and suppression of inflammatory burden, likely explain the parallel trajectories of vascular, cognitive, and sensory aging. The preservation of cochlear microcirculation may thus represent part of a unified, system-level mechanism through which flavonoids exert geroprotective effects across multiple organ systems [34]. Building on this mechanistic insight, incorporating flavonoid-centered nutritional recommendations into dietary guidelines may provide dual benefits for cardiometabolic and neuro-sensory health, supporting the emerging paradigm of whole-body vascular aging [35,36].

Several limitations warrant consideration. Flavonoid intake was estimated from self-reported dietary assessments, which may underestimate actual intake due to recall errors and variations in day-to-day intake. Although we adjusted for major lifestyle and metabolic covariates, residual confounding from genetic factors, environmental noise, or exposure to ototoxic medications cannot be excluded. Additionally, hearing loss ascertainment relied on linked records and self-reported data rather than audiometric testing, which may result in nondifferential misclassification. The mediation analysis was cross-sectional in biomarker timing, and thus, causal inference should be made cautiously. Future studies incorporating repeated biomarker measurements and objective auditory thresholds would help clarify temporality and dose–response relationships.

Despite these limitations, this study offers novel population-level evidence that a flavonoid-rich dietary pattern is associated with a lower risk of hearing loss, partly mediated by systemic inflammation. Clinically, this underscores the potential for nutritional interventions to complement conventional auditory care and extend preventive strategies into the domain of dietary behavior. Our findings underscore the clinical relevance of dietary composition in maintaining sensory health and provide a foundation for integrating nutritional interventions into strategies for preventing hearing loss. Encouraging population-wide consumption of flavonoid-rich foods could represent a simple, scalable, and sustainable approach to promote healthy aging and preserve cognitive and sensory functions throughout life. Furthermore, future interventional or longitudinal metabolomic studies should investigate whether modulation of inflammatory pathways through flavonoid supplementation or dietary counseling can lead to measurable preservation of hearing capacity in older adults.

## 5 Conclusions

This large prospective cohort study demonstrated that greater adherence to a flavonoid-rich dietary pattern, represented by a higher Flavonoid Diet Score, was independently associated with a lower risk of incident hearing loss. The association persisted after adjustment for demographic, lifestyle, and cardiometabolic factors, and was partially mediated by systemic inflammation indexed by C-reactive protein, supporting an anti-inflammatory pathway.

These findings highlight the potential of dietary flavonoids as modifiable nutritional factors that may contribute to the preservation of auditory function and the prevention of sensory aging. From a public health perspective, promoting regular consumption of flavonoid-rich foods such as berries, citrus fruits, green tea, and dark-colored vegetables could represent a simple, accessible, and low-cost strategy to reduce the burden of hearing loss in aging populations.

Further longitudinal and intervention studies are warranted to clarify dose–response relationships and to explore whether dietary modification targeting flavonoid intake can effectively preserve auditory health and delay age-related sensory decline.

## Authors’ contributions

Y.H. conceptualized the study, performed the statistical analyses, and drafted the manuscript. K.Y.L. contributed to the interpretation of data and clinical contextualization. I.S. supervised the project and critically revised the manuscript for important intellectual content. D.J.J. contributed to study design, data interpretation, and manuscript revision. All authors reviewed and approved the final version of the manuscript.

## Consent for publication

Not applicable.

## Ethics approval and consent to participate

The UK Biobank study was approved by the National Research Ethics Service Committee North West–Haydock (Ref: 11/NW/0382). All participants provided written informed consent prior to participation.

## Declaration of Generative AI and AI-assisted technologies in the writing process

During the preparation of this work the authors used ChatGPT (AI language model) in order to help with grammar, spelling, and sentence structure. No analytical or interpretative content was produced by the model. After using this model, the authors reviewed and edited the content as needed and take full responsibility for the content of the publication.

## Funding

This research was supported by a grant of the Korea Health Technology R&D Project through the Korea Health Industry Development Institute (KHIDI), funded by the Ministry of Health & Welfare, Republic of Korea (RS-2022-KH130590).

## Availability of data and materials

The data that support the findings of this study are available from the UK Biobank (www.ukbiobank.ac.uk) under Application Number 146430, subject to institutional approval and data access policies.

## Declaration of competing interest

The authors declare that they have no competing interests.
