# Supplementary material for: Flavonoid-rich dietary patterns and the risk of incident hearing loss: evidence from the UK Biobank cohort
Source: J Nutr Health Aging. 2026 Jun 3;30(7):100891. doi: 10.1016/j.jnha.2026.100891 (PMC13255062; doi:10.1016/j.jnha.2026.100891)
Supplement: Supplementary file 1 [file mmc1.docx]

**Supplementary table 1. Components and Scoring of the Flavonoid diet Score (FDS)**

| Component | Category | Standard serving (Oxford WebQ) | Rationale for inclusion | Range (servings/day)* |
| --- | --- | --- | --- | --- |
| Black tea | Beverage | 250 mL cup | Major source of flavan-3-ols and polymers | 0–4 (capped) |
| Green tea | Beverage | 250 mL cup | Source of catechins and flavan-3-ols | 0–4 (capped, combined with black tea) |
| Red wine | Beverage | 125 mL glass | Major contributor to anthocyanin intake | 0–3 |
| Apples | Fruit | 1 medium apple (~150 g) | Source of flavonols and flavan-3-ols | 0–3 |
| Berries | Fruit | 1 serving (~80 g) | Source of anthocyanins | 0–3 |
| Grapes | Fruit | 1 serving (~80 g) | Source of anthocyanins and flavan-3-ols | 0–3 |
| Oranges (incl. satsumas) | Fruit | 1 medium (~120 g) | Source of flavanones | 0–3 |
| Grapefruit | Fruit | 1 serving (~120 g) | Source of flavanones | 0–3 |
| Sweet peppers | Vegetable | 1 serving (~80 g) | Source of flavones | 0–3 |
| Onions | Vegetable | 1 serving (~80 g) | Source of flavonols | 0–3 |
| Dark chocolate† | Snack | 1 serving (~25 g) | Source of flavan-3-ols (low intake in this cohort) | 0–2 |
